# Native learning ability and not age determines the effects of brain stimulation

**DOI:** 10.1038/s41539-024-00278-y

**Published:** 2024-11-27

**Authors:** Pablo Maceira-Elvira, Traian Popa, Anne-Christine Schmid, Andéol Cadic-Melchior, Henning Müller, Roger Schaer, Leonardo G. Cohen, Friedhelm C. Hummel

**Affiliations:** 1 Defitech Chair for Clinical Neuroengineering, Neuro-X Institute (INX), École Polytechnique Fédérale de Lausanne (EPFL), Geneva, Switzerland; 2 Defitech Chair for Clinical Neuroengineering, Neuro-X Institute (INX), EPFL Valais, Clinique Romande de Réadaptation, Sion, Switzerland; 3 Wyss Center for Bio- and Neuroengineering, Geneva, Switzerland; 4 University of Applied Sciences Western Switzerland (HES-SO), Valais-Wallis, Switzerland; 5 Human Cortical Physiology and Neurorehabilitation Section, NINDS, NIH, Bethesda, MD USA; 6 Clinical Neuroscience, University of Geneva Medical School, Geneva, Switzerland

**Keywords:** Cognitive ageing, Learning and memory, Motor cortex, Sensory processing

## Abstract

Healthy aging often entails a decline in cognitive and motor functions, affecting independence and quality of life in older adults. Brain stimulation shows potential to enhance these functions, but studies show variable effects. Previous studies have tried to identify responders and non-responders through correlations between behavioral change and baseline parameters, but results lack generalization to independent cohorts. We propose a method to predict an individual’s likelihood of benefiting from stimulation, based on baseline performance of a sequential motor task. Our results show that individuals with less efficient learning mechanisms benefit from stimulation, while those with optimal learning strategies experience none or even detrimental effects. This differential effect, first identified in a public dataset and replicated here in an independent cohort, was linked to one’s ability to integrate task-relevant information and not age. This study constitutes a further step towards personalized clinical-translational interventions based on brain stimulation.

## Introduction

The acquisition of sequential motor skills is essential for the completion of activities of daily living. When encountered for the first time, the execution of a sequential motor task entails a speed-accuracy tradeoff^1^, in which performing the task at higher speeds often leads to a drop in accuracy. With practice, individuals experience a shift in this tradeoff^2,3^, allowing for an accurate execution of the task at increasing speeds. Optimal motor skill acquisition, characterized by the aforementioned shift in the speed-accuracy tradeoff occurring at the early stages of training, depends on prioritizing the improvement of the accuracy over that of the speed^4^.

The acquisition of sequential motor tasks is often less efficient in older adults, who frequently depict an overall lower performance in the execution of such tasks^4–7^. As such, the plasticity-augmenting properties of non-invasive brain stimulation (NIBS)^8^, constitute a promising option to improve motor skill acquisition in older adults^9^. Notably, anodal transcranial direct current stimulation (atDCS) applied over the motor cortex can improve motor skill acquisition in older adults^7,10,11^ by accelerating the shift in the speed-accuracy tradeoff, streamlining the acquisition of sequential motor tasks^4^. This effect of atDCS appears to be reserved to the early stages of training (i.e., mostly to the first training session), and exclusive to individuals with age-related diminished learning abilities^4^. However, some previous studies have shown beneficial effects of atDCS in young adults as well^12–15^. This raises the question of what are the determinant factors behind an individual’s response to a NIBS intervention, such as atDCS, possibly accounting for the large variability in its effects on motor skill acquisition^11,12^. Previous studies have suggested a differential response to stimulation may be related to previous training or experience, with absent^16^ or even deleterious effects^17^ on highly trained individuals.

Here, we studied the effects of atDCS applied concomitant to motor training in middle-aged and older adults over the course of ten days. Using a public dataset^4^, we constructed a machine-learning model to classify individuals into different categories of optimal and suboptimal learners, and applied it to the dataset acquired for this study. With this model, we estimated each individual’s ability to acquire a sequential motor task, as well as their likelihood to benefit from atDCS, based on their initial performance in this task. Our results suggest a differential effect of atDCS, granting larger benefits to individuals with less efficient learning mechanisms while being detrimental to individuals possessing optimal learning strategies. These effects were neither necessarily characteristic of nor reserved to individuals of a certain age.

## Results

### A group of uncharacteristically efficient, healthy older adults

We conducted a study to assess the effects of applying atDCS over the left-hand representation of the primary motor cortex (M1) during the acquisition of a well-established finger-tapping task^4,7,15,18,19^. Building on our previous findings^4^, which showed an effect of atDCS in healthy older adults, we recruited cohorts of middle-aged (50–65 y/o; *n* = 20, 11 female; age *μ* = 59.05 y/o) and older adults (>65 y/o; *n* = 20, 10 female; age *μ* = 71.7 y/o), to search for additional effects of stimulation related to a higher stimulation dose (i.e., 10 days instead of five). The participants received either verum stimulation (i.e., active; middle-aged = 10, age *μ* = 58.9; older = 10, age *μ* = 71.4) or placebo stimulation (middle-aged = 10, age *μ* = 59.2; older = 10, age *μ* = 72.1) for 20 minutes daily, as they practiced the finger-tapping task using their left hand. All participants were right-handed and did not have any previous training as musicians, stenographers, or comparable activities requiring high dexterity. The task consisted of replicating an explicitly shown, nine-digit numerical sequence as fast and as accurately as possible using four buttons. We conducted a series of analyses following the same analytical pipeline as we used before^4^, looking for effects of atDCS on the execution of the task in both age groups. Please refer to the Supplementary Notes2–5, and Supplementary figs. 1–4 for a detailed account of these analyses. Overall, we did not find evidence for an effect of stimulation in middle-aged adults, while we found an effect leading to similar accuracy dynamics in the older adults receiving verum stimulation, matching our previous reports^4^. However, we noticed the older adults receiving the placebo showed much better performance at baseline compared to the verum group of older adults, with their general performance being similar to that of middle-aged adults. These results challenged our previous observations of older adults being less efficient in the acquisition of the finger-tapping task^4^, as well as previous studies showing diminished motor performance in older individuals^5,6,20^. With this in mind, we implemented an algorithm to determine an individual’s efficiency at acquiring the finger-tapping task, with the goal of assessing whether the state of the neural system supporting the acquisition of this task had an impact on the effect of stimulation. In the analyses to follow, the previously described dataset (i.e., including 20 middle-aged and 20 older adults) will be referred to as the “Validation dataset”.

### The likelihood to benefit from stimulation

Our working hypothesis is that the optimal acquisition of the finger-tapping task relies on prioritizing the improvement of the accuracy at the early stages of training. Under this hypothesis, we would consider individuals reaching a plateau in accuracy on the first training session to be “optimal learners”, while those reaching a plateau at a later stage would be considered “suboptimal”. Further, based on our previous findings^4^, we propose atDCS possesses a restorative rather than an enhancing quality. As such, we would expect optimal learners not to benefit from atDCS, while suboptimal learners would profit from stimulation, with larger boons expected for worse learners. In regard to the prioritization of accuracy over speed, we cannot entirely rule out the possibility of it being a matter of personal preference. However, the marked accuracy dynamics related to the application of atDCS suggest this process may be driven by the system’s ability to manage neural resources, with the goal of maximizing performance while minimizing cognitive load^21^.

**Fig. 1:**
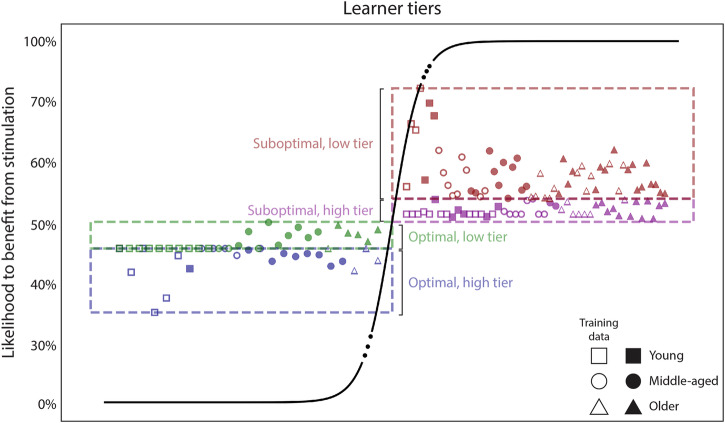
The likelihood of an individual’s response to stimulation. We trained a classifier to discriminate between optimal and suboptimal learners, using data from unstimulated participants in the Modeling dataset. Then, we extracted the distance between each individual and the hyperplane separating both classes of learners and projected it onto a sigmoid function to generate a map of probabilities. Those above 50% belong to the “suboptimal” class, thus expected to have a chance to benefit from stimulation. Each class is further divided into two segments (i.e., tiers), based on the median split of the probabilities from the training data. The markers are used to encode the age group of each participant, as well as whether each particular data point belongs to the training dataset (hollow markers) or not. Of note is that each tier contains individuals from three age groups (i.e., young, middle-aged, and older). Please note that there is no meaning to the *x* axis, so the age clusters forming along the horizontal axis bear no meaning. For details on the individual predictions (i.e., likelihood, learner group, etc.) please refer to the data repository of the present work.

Following this reasoning, we implemented an algorithm to determine the likelihood of an individual reaching a plateau in accuracy on the first day of training based on baseline parameters. Specifically, we trained a classifier to label individuals as either “optimal” or “suboptimal” learners based on their age, and measures of their initial speed and initial accuracy (please refer to Methods for details on the features drawn). To train the classifier, we used a previously published dataset^4^, which we will dub the “Modeling dataset” henceforth. We trained the classifier with both unstimulated or placebo-stimulated participants from the Modeling dataset, using 80% of the data for training and 20% for testing, achieving a prediction accuracy of 97% (i.e., F1 score). Please note that we intentionally used all the aforementioned data for training the model (without leaving a validation set aside), so this score only indicates the separability of the training features. After training the classifier, we projected the predictions from the model onto a sigmoid function (i.e., logistic function) to estimate the likelihood of each individual reaching a plateau on the first training day. Figure 1 illustrates the logistic function, with values above 50% classified as “Suboptimal” (i.e., with above-chance likelihood to benefit from stimulation). After projecting the model predictions onto the logistic function, we divided each class into a high and a low tier, based on the median split of the probabilities. The data shown in Fig. 1 contains both the training data (hollow markers), and the testing and validation data that will be detailed later. Please refer to Methods for more details on this model, and to the data repository to find the likelihoods estimated for each individual.

### The differential effect of atDCS across learner tiers

**Fig. 2:**
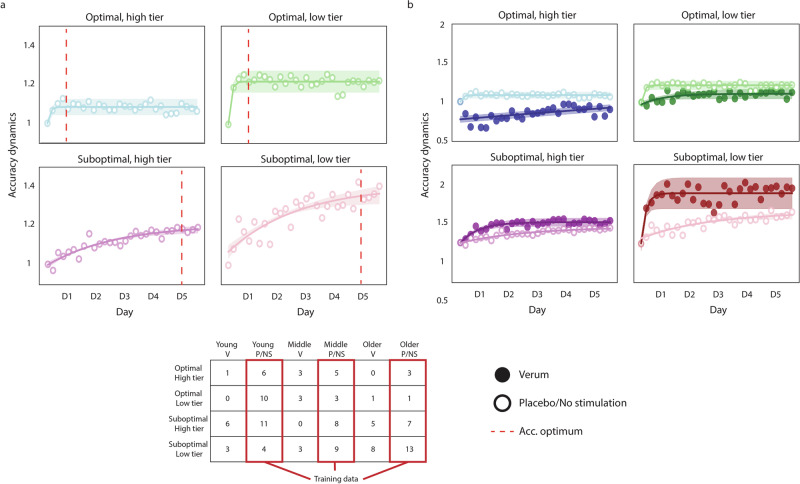
Accuracy dynamics in optimal and suboptimal learners of the Modeling dataset. **a** Accuracy dynamics in the training dataset, illustrating the efficacy of the classifier, as optimal learners reach an accuracy optimum on the first training day, while suboptimal learners do so towards the end of training (red dashed line). **b** Accuracy dynamics in all groups of the Modeling dataset, contrasting the groups receiving verum stimulation to those receiving no stimulation (i.e., those shown in **a**). The results show a differential effect of stimulation, in which the benefit of stimulation is more pronounced in less efficient learners, while being detrimental to optimal learners. The accuracy dynamics are computed as the ratio of the accuracy of each training block to the accuracy of the first training block, reflecting the performance change relative to the first block. In all plots, the markers represent the average accuracy of each training block (i.e., 6 blocks per day), and the shaded regions reflect the 95% confidence interval of the fitted lines. The hollow markers correspond to groups receiving either no stimulation or placebo stimulation, while the full markers correspond to the groups receiving verum stimulation. The table depicts the number of individuals assigned to each learner tier, and highlights the groups used to train the classifier (in red). In the table, each column corresponds to young, middle-aged, and older adults receiving verum (V), placebo (P), or no stimulation (NS).

The model depicted in Fig. 1 provides a continuous range for the likelihood of a person to benefit from stimulation. We expected a differential effect of stimulation across the four learner tiers, with larger effects present in the lower tier of suboptimal learners and no effect in both tiers of optimal learners. The performance of the finger-tapping task can be characterized in terms of the speed and the accuracy of execution. In the past, we found atDCS to have an effect on the accuracy dynamics, leading to the rapid optimization of this parameter, while not finding an effect in speed^4^. To simplify the presentation of the results, we will focus on the accuracy dynamics for most of the comparisons; please see Supplementary Note *8* to find the speed dynamics of each group. Figure 2a shows the accuracy dynamics in each learner tier of the training data (i.e., participants receiving either placebo stimulation or no stimulation whatsoever, from the Modeling dataset). The depicted results illustrate the classifier captured the behavior we intended, with optimal learners reaching a plateau in accuracy on the first training day, and suboptimal learners doing so towards the end of training. Please note that the learner tiers derived from the probability map we built show a gradient: optimal learners reach a plateau on the first day (as enforced by the classifier), but the high tier describes a smaller relative change, suggesting they were closer to the optimum than the low tier. On the other hand, both tiers of suboptimal learners reach a maximum towards the end of training, with the low tier experiencing a larger improvement over the course of practice. Figure 2b shows the same unstimulated groups (i.e., no stimulation and placebo) shown in Fig. 2a, but contrasted to the participants that received verum stimulation in the Modeling dataset. Please note a change in scale was necessary to show the dynamics in the verum groups.

The results show a differential effect of stimulation, in which the low tier of suboptimal learners describes a sharp increase in accuracy on the first training session and reaches a plateau. This effect was less pronounced in the high tier of suboptimal learners, but the change in accuracy was significantly higher in the low tier of suboptimal learners (*t*_44_ = 2.223, *d* = 1.833, *P* = 0.03). In contrast, both tiers of optimal learners seem to perceive deleterious effects of stimulation; while those in the low tier of optimal learners were not significant, the accuracy of the high tier of optimal learners worsened significantly on the first day (*t*_20_ = 3.887, *d* = 4.39, *P* = 0.0009). Please note that none of the data belonging to the verum groups was used to fit the model described previously; the model was only applied to predict the individual likelihoods in these groups.

### Validation of the differential effect of stimulation

**Fig. 3:**
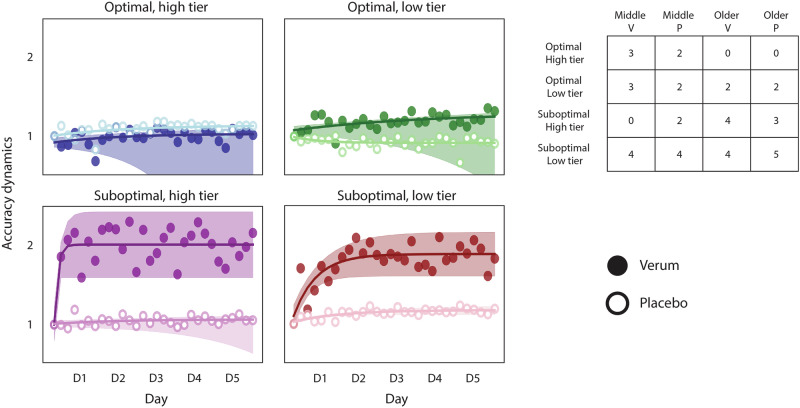
Accuracy dynamics in the validation dataset. Accuracy dynamics in an independent dataset including middle-aged and older adults, receiving either verum or placebo stimulation. The results confirm the differential effect of stimulation observed in the Modeling dataset, in which atDCS benefits suboptimal learners only. The detriment seen in the groups of optimal learners is less apparent; indeed, the low tier does not appear to be affected. Please keep in mind some groups have a low number of individuals, which results in a widespread confidence interval. The accuracy dynamics are computed as the ratio of the accuracy of each training block to the accuracy of the first training block, reflecting the performance change relative to the first block. In all plots, the markers represent the average accuracy of each training block (i.e., 6 blocks per day), and the shaded regions reflect the 95% confidence interval of the fitted lines. The hollow markers correspond to groups receiving placebo stimulation, while the full markers correspond to the groups receiving verum stimulation. The table depicts the number of individuals assigned to each learner tier, with each column corresponding to middle-aged and older adults receiving verum (V) or placebo (P) stimulation.

We applied the model to the Validation dataset mentioned previously, including 20 middle-aged and 20 older adults. Same as before, these data were kept separate from the training data, and we used the model to predict the likelihood to benefit from stimulation for each participant and to assign them to one of the four learner tiers. Figure 3 shows the accuracy dynamics for this new dataset.

**Fig. 4:**
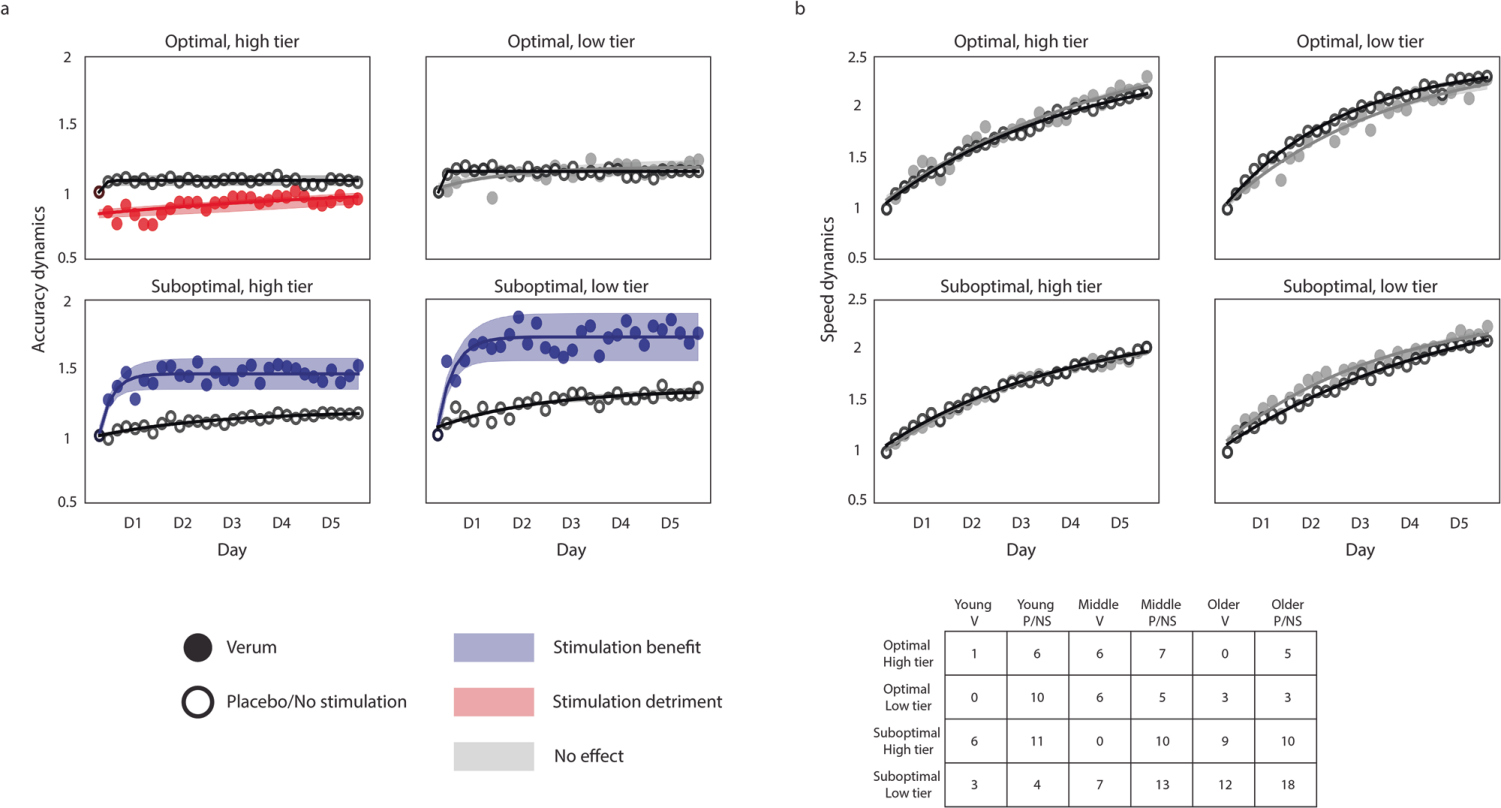
Accuracy and speed dynamics for the combined datasets. The data depicted combines the Modeling and the Validation datasets, so each group contains individuals from all age groups. **a** Accuracy dynamics of the combined datasets, showing a clear differential effect of atDCS, detrimental to optimal learners and beneficial to suboptimal learners. The benefit of stimulation is most pronounced in the least efficient individuals (i.e., the low tier of suboptimal learners). The verum groups are colored to code stimulation benefits (blue), detriments (red), or a lack of effect (gray). **b** Speed dynamics in all learner tiers, with patterns being similar between stimulation conditions. Please note that the consistent improvement in speed (paired with the maintenance of accuracy) suggests a consistent improvement in performance throughout training, which does not support the notion of a ceiling effect in the performance of the task. The dynamics (both for accuracy and speed) are computed as the ratio of the score of each training block to the score of the first training block, reflecting the performance change relative to the first block. In all plots, the markers represent the average accuracy (**a**) and speed (**b**) of each training block (i.e., 6 blocks per day), and the shaded regions reflect the 95% confidence interval of the fitted lines. The hollow markers correspond to groups receiving either no stimulation or placebo stimulation, while the full markers correspond to the groups receiving verum stimulation. The table depicts the number of individuals assigned to each learner tier, and each column corresponds to young, middle-aged, and older adults receiving verum (V), placebo (P), or no stimulation (NS).

The results show a similar effect of stimulation in both tiers of suboptimal learners. The detrimental effect observed previously in optimal learners is less apparent, although the verum group of the high tier appears to be slightly worse than the placebo. The large variability present in some of the groups (e.g., the verum group in the optimal, high tier) is probably derived from the reduced number of individuals constituting them, which complicates the interpretability of the statistical analysis for these groups. However, the differential effect of stimulation is largely preserved in the Validation dataset. Indeed, if we pool the Modeling and Validation datasets, the differential effect of stimulation becomes much clearer. Figure 4 shows the accuracy and the speed dynamics for both datasets. In this case, we chose to show the speed dynamics as well to show that the participants’ performance continues to improve throughout training, which argues against the possible interpretation of the accuracy plateau being the result of a ceiling effect. Please see Supplementary fig. *7* to find the separate speed dynamics for the Modeling and the Validation datasets. Please note that none of the data from the verum groups in either dataset was used to train the model.

**Fig. 5:**
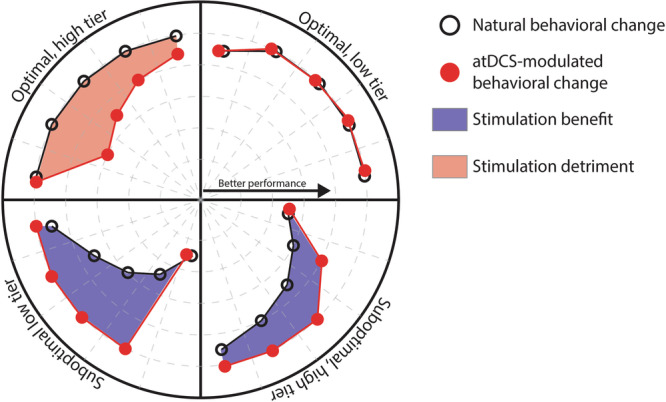
Main finding. Anodal transcranial direct current stimulation (atDCS), applied over the hand representation of the motor cortex concomitant to the training of a sequential motor sequence, has differential effects as a function of the recipient’s ability to integrate task-relevant information at the early stages of training. Stimulation benefits individuals with seemingly less efficient learning mechanisms, enabling the rapid optimization of the accuracy of execution of the motor sequence. In contrast, individuals possessing optimal learning mechanisms experience detrimental effects of stimulation, leading to drops in the accuracy of execution.

Our results confirmed the differential effect of stimulation we had hypothesized, with more pronounced effects in the low tier than in the high tier of suboptimal learners. The sharp improvement in accuracy we have proposed to be derived from the application of atDCS was present in both groups of suboptimal learners, supporting our previous findings on the effects of atDCS in the acquisition of the finger-tapping task^4^. The change in accuracy in the low tier of suboptimal learners was significantly higher in the verum group than in the placebo/unstimulated group (*t*_63_ = 2.365, *d* = 1.7, *P* = 0.02) on the first day of training, and kept being significantly higher until day 3. For the high tier of suboptimal learners, the change in accuracy was significantly higher for the verum group on the first day (*t*_61_ = 3.716, *d* = 2.08, *P* = 0.0004), and remained being significantly higher throughout training. Please note that while these two categories have a majority of individuals over the age of 65 (i.e., older adults), there are both young and middle-aged adults in both categories of suboptimal learners. In regards to both categories of optimal learners, our expectation of having little or no effect of stimulation was met by the low tier of optimal learners, for which the dynamics appear to be quite similar. In contrast, individuals in the high tier of optimal learners worsened significantly at the early stages of training (*t*_29_ = 3.612, *d* = 2.884, *P* = 0.0011), suggesting a detrimental effect of stimulation for individuals with optimally functioning neural systems. This differential effect is summarized in Fig. 5.

### Estimated likelihood to benefit from stimulation and cognitive functions in middle-aged and older adults

We used the Mini-Mental State Examination (MMSE^22^) as an inclusion criterion for middle-aged and older adults volunteering for this study (>25/30), in order to assert the preservation of general cognitive functions in these groups. Considering our results, suggesting individuals possessing less efficient learning mechanisms are more likely to benefit from stimulation, we tested the relationship between the estimated likelihood to benefit from stimulation and the MMSE scores in middle-aged and older adults. However, we did not find a significant correlation between these two parameters. Please refer to Supplementary Note 9, and see Supplementary fig. 8 to find the MMSE scores for all middle-aged and older participants, visualized against their estimated likelihood to benefit from stimulation.

### Electrophysiology

We used the SICI paradigm^23–25^ of transcranial magnetic stimulation (TMS) to quantify GABAergic intracortical inhibition within M1, and to assess any changes related to the application of stimulation after the ten training days in the participants of the Validation dataset. We did not find statistically significant changes in the SICI recordings performed at rest in any of the groups, providing no evidence for neither training-derived nor stimulation-derived effects. The modulation of SICI, assessed by applying this paradigm during movement preparation (please refer to the Methods for more details), changed significantly in the middle-verum group (*t*_27_ = 3.01, *d* = 1.56, *P* = 0.005), and was significantly higher than in the middle-placebo group (*t*_27_ = 3.65, *d* = 1.95, *P* = 0.001). In older adults, there was no significant difference between verum and placebo (*F*_1_ = 3.61, *η*^2^ = 0.1, *P* = 0.06), and no evidence for a significant change after training (*F*_1_ = 0.46, *η*^2^ = 0.01, *P* = 0.5). Please see Supplementary Note *7* and Supplementary fig. 6 for more details on these measurements.

## Discussion

The present work adds to the understanding of the differential, individual effect of atDCS on the acquisition of a previously unknown sequential motor task, which was more pronounced in individuals with less efficient learning mechanisms while being detrimental to those expected to learn the task most efficiently.

Previous research shows atDCS can improve motor skill acquisition, but the direction and magnitude of this effect varies among reports, and is difficult to predict for each individual^11,12^. Therefore, we implemented a machine-learning method to label individuals as either optimal or suboptimal learners and studied the effect of stimulation in each of the resulting categories. Our results showed the least capable learners among our cohorts to benefit from stimulation to the largest extent, with this benefit being less pronounced as initial skill levels increased. At the other extreme of the spectrum, the most skilled learners showed even detrimental effects of atDCS, suggesting an interfering effect of stimulation on optimal neural systems.

Even though the overall effect of atDCS on the acquisition of sequential motor tasks seems to be predominantly positive^12^, the variability in reported effects across motor and cognitive domains is high^26^. There are multiple potential sources of this variability^27,28^, and there have been various attempts at identifying subgroups of responders and non-responders^29,30^. Subgroup analyses can lead to a biased estimate of the effects of stimulation^31^, but they can also provide hints as to which parameters can be determinant of an individual’s response to any given intervention. The identification of biomarkers reflecting the state of the neural system can then be used to narrow the administration scope of atDCS and other comparable techniques, which may lead to a generalized increased efficacy in clinical applications^32^. Previous research has shown a relationship between the post-intervention performance and different baseline parameters, such as initial training levels^16,17^ or the magnitude of induced electric fields^33^. These associational approaches are highly valuable in the identification of likely differentiating aspects to consider when applying tDCS, but their practical use is limited, as the insights provided by them cannot be directly generalized to other samples.

In contrast, the present approach consists of predictive modeling. This method uses an independent dataset acquired for a different study and constructs a model to estimate the native state of the neural system. This estimation is done indirectly, and it depends on the relative performance of each individual in the first block of training with respect to the baseline block. The difference in performance between these two blocks may indicate the ability of an individual to rapidly integrate task-relevant information. As each of these two blocks consists of a different numerical sequence, the information being integrated is likely universal to the task and sequence-unspecific. In the context of the sequence-tapping task, this information may consist of the mapping of the numbers to the fingers and the physical properties of the interface used to perform the task (i.e., the keys). In the context of the present work, this performance change between blocks would be an indirect biomarker for the state of the neural components supporting the early stages of skill acquisition. Please note that upon completion of the classification model construction, the dataset acquired in this study has not been “seen” by the model, so there is no bias in the class prediction.

In regards to the observed performance within each learner class, the results seen in both groups of suboptimal learners and in the lower tier of optimal learners were expected from our hypothesis. The presence of larger effects in less optimal individuals can be equated to the more pronounced effects seen in more affected aging^34^ and clinical^35–37^ populations. However, as previously explained, the results obtained with our method go beyond an association among these variables.

The apparent absence of an effect in the lower tier of optimal learners could be attributed to a ceiling effect for skilled individuals in this task^17^. However, the monotonically increasing speed and the fact that the accuracy in most participants does not stabilize at 100% (please see Supplementary fig. *2*) suggests this is not the case. The detrimental effect observed in the higher tier of optimal learners has been observed in highly trained individuals (e.g., in pianists^17^), and could be related to the additional energy input disrupting an already optimal system^38^. Please note that in this last group, none of the participants had prior extensive training in tasks requiring high dexterity, and the group contained individuals of all age groups, which does not support the interpretation of these effects being related to prior expertize^17^.

In sum, even though previous research has shown varying effect magnitudes related to baseline levels, this approach is, to our knowledge, the first to offer a quantitative prediction of the likelihood of an individual to respond to stimulation, with a differential effect present in the overall performance dynamics and not on single time-point averages.

atDCS is assumed to facilitate the firing of neuronal populations within the stimulated area by altering the neurons’ resting membrane potential, effectively lowering the neuronal threshold and thus increasing the likelihood of neurons depolarizing^39^, which results in an increased cortical excitability^40^. Further, pharmacological studies^41^ have revealed a decrease in GABAergic intracortical inhibition resulting from the application of atDCS^42^, thought to induce long-term potentiation- (LTP-) like plasticity^43^. However, we did not find such a change, as measured by the SICI paradigm; this matched previous reports, in which no change in GABAergic intracortical inhibition was observed when atDCS was applied concomitantly to motor training^4,44^. An interpretation for the absence of such a change could be that LTP-like plasticity is not being induced within M1 or, like Amadi and colleagues proposed^44^, that the LTP induced by atDCS competes with the one induced by motor training itself, leaning towards a state of long-term depression (LTD). If this latter interpretation were correct and the LTP-like plasticity-inducing effect of both processes canceled out, it could explain why we do not see any effects of stimulation on the actual execution of the movements (i.e., on the speed). In this case, the question remains: where does the observed effect on the accuracy come from?

We have proposed that atDCS streamlines the transmission of task-relevant information upstream within the motor network. This seems to be limited to the early stages of acquisition, which is assumed to be dominated by processes involving the mapping of stimuli to responses and the storage of spatial coordinates in memory. These processes would be essential to the construction of internal models, which consist of neural constructs that can simulate natural processes^45,46^. The concept of internal models is often split into two categories: forward models, used to predict future system states, and inverse models, estimating the system commands leading to a specific state^45^. In the context of motor learning and motor control, forward models are thought to enable the generation of timely actions that would otherwise be impossible due to time delays inherent to the neural system^47^. Further, they are thought to drive motor adaptation based on the error between the model’s predicted and the system’s measured sensory feedback^48^. On the other hand, inverse models contain functions mapping a set of motor outputs to a certain objective, which makes their construction and deployment essential to the acquisition of new skills. Being so that our model can discriminate optimal from suboptimal learners (as per the definition used in the present work) based on their performance on two different motor sequences, an interpretation could be that atDCS is improving the construction of inverse internal models. Multiple studies suggest internal models are stored in the cerebellum^49–51^. However, some studies propose the cerebellum contains exclusively forward internal models^52,53^, while inverse internal models may be stored in the motor cortex^54–56^. The separate allocation of forward inverse models was evaluated using atDCS applied to the cerebellum^57^. Here, we did not test for this distinction explicitly, but the differential effect of stimulation observed at the early stages of training would support the notion of inverse internal models residing in M1. The mechanism through which the emergence of these models could be streamlined is not clear, but being so that we did not find evidence of LTP-like plasticity being induced at M1, a possibility could be that atDCS is improving the transmission of information to other structures within the motor network. Harvey and Svoboda^58^ showed that a “crosstalk” effect exists, in which LTP at one synapse influences the plasticity threshold at neighboring synapses. Depending on the stage of acquisition, this threshold shift could result in LTP being induced at synapses leading upstream of the motor network; with stimulation acting on metaplasticity (i.e., altering the “plasticity of synaptic plasticity”^59^), it could facilitate the relay of information and prepare the system to encode the incoming information more efficiently^60^. Please note that in Harvey and Svoboda’s study^58^, subthreshold stimulation did not induce LTP; it was only when combined with the LTP crosstalk from the suprathreshold stimulation that synaptic efficiency increased. In the context of our study, it would be through the combination of atDCS (which is subthreshold) and motor training-derived changes in synaptic efficiency, that LTP would be induced. The need for this aggregated interaction would also explain why the induced electric field, spanning across a broad area, has such specific effects.

The differential effect of atDCS could then be related to the native metaplastic properties of the motor network. Aberrant metaplasticity has been related to diminished learning abilities, with its emergence being associated with substance addiction (e.g., ethanol^61^) and neurological disorders (e.g., dystonia^60^). As such, atDCS could help restore metaplasticity to a more normal state. On the other side of the spectrum, the most efficient learners, possibly possessing highly efficient brain networks, could be driven off balance by the external input.

Non-invasive brain stimulation shows promise for the enhancement of failing motor^7,9^ and cognitive^62–64^ functions in healthy older individuals, thus constituting an exciting option for therapeutic and rehabilitative applications^65–67^. However, the corpus of literature involving the use of NIBS for this intent reports highly variable results^11^, obstructing the quantification and estimation of the potential benefits of these techniques. The present study provides an illustration of what the deployment of stimulation techniques such as atDCS could look like in a clinical setting. In such a scenario, healthcare providers could assess the likelihood of an individual benefiting from stimulation based on a set of parameters related to the mechanism to be treated (e.g., rehabilitation, memory), which will necessitate the prior identification of such parameters. Here, we have identified a small set of parameters that may jointly represent the ability of an individual to integrate task-relevant information in a well-delimited context, constituted by the finger-tapping task. However, additional investigations are necessary to identify parameters that may relate to other aspects of behavior and cognitive functions, and that may be influenced by brain stimulation such that these techniques may be used to their full potential for a personalized therapeutic approach. Future endeavors may benefit from a more detailed characterization of individual cognitive functions based on a more extensive neuropsychological assessment in order to better capture individual functions. In turn, this characterization may allow for better contrast and understanding of the models’ parameters, essential for a more general application of predictive methods in real-life scenarios.

## Methods

### Participants

The present work includes data from a total of 153 healthy volunteers. Forty healthy adults volunteered specifically for this study (“Validation set”), while the rest were volunteers of previous studies (^4^, “Modeling set”). In this study, we grouped the participants according to their age, as middle-aged (50–65 y/o; *n* = 20, 11 female; age *μ* = 59.1 y/o), and older (>65 y/o; *n* = 20, 10 female; age *μ* = 71.7 y/o) adults. They were randomly assigned within each age group to receive either real stimulation (i.e., verum; middle-aged = 10, age *μ* = 58.9; older = 10, age *μ* = 71.4) or placebo stimulation (middle-aged = 10, age *μ* = 59.2; older = 10, age *μ* = 72.1). The in-depth analyses we implemented for the characterization and classification of individuals as a function of their initial performance in the finger-tapping task was built on the data belonging to the Modeling set. These participants were also grouped according to their age as young (18–30 years old; *n* = 41, 27 female; age *µ* = 24.5 years old), middle-aged (50–65 years old; *n* = 34, 20 female; age *µ* = 57.7 years old), and older (>65 years old; *n* = 38, 21 female; age *µ* = 72.3 years old) adults.

All participants were right-handed, confirmed using the Edinburgh Handedness Inventory^68^. The participants reported not having a previous history of serious medical conditions (General Health Questionnaire, GHQ) or contraindications for tDCS (questionnaire based on safety recommendations for these techniques^69,70^. We performed a neurological examination on all middle-aged and older participants to ensure they were healthy, and performed the Mini-Mental State Examination (MMSE^22^) to verify that all middle-aged and older participants scored at least 26 out of 30 points. None of the participants had extensive prior experience in tasks requiring high dexterity, such as playing music, stenography, videogames, etc. All participants gave their written informed consent under protocol guidelines approved by the Cantonal Ethics Committee Vaud, Switzerland (project No. 2017-00301), according to the Declaration of Helsinki. All participants completed training except for one older adult, who chose to leave the study after six days of training for finding the steps involved in setting up the stimulation and motor training to be too overwhelming. No serious adverse events were reported, and only three participants reported mild adverse events (please see Supplementary Note 11).

### Motor training

We used the same finger-tapping task as in our previous study^4^, requiring participants to replicate a nine-digit sequence as fast and as accurately as possible (Fig. 5a). The participants trained their left (non-dominant) hand for 20 min each day for two sets of five consecutive days (i.e., total ten days), with two rest days in between (Fig. 5b). We asked participants to train using their non-dominant hand to present a relatively higher challenge, in order to better observe the gradual improvement of different aspects of performance (e.g., speed and accuracy) in this task. Each training session consisted of six 90-second training blocks and one 90-second “catch block” (presenting a sequence different from the training sequence) halfway through training to test for sequence-unspecific learning. Between blocks, participants were allowed to rest for 90 seconds. On the first training day, participants performed a 90-second “Baseline block” without atDCS, meant to assess individual skill levels before training. The Baseline block contained a sequence that was different from those presented during the training blocks and the catch blocks.

### Electrical stimulation of M1 during training

We applied stimulation using the setup we described in an early report of this study^71^, with the anode and the cathode placed at the C4 and FP1 locations, respectively, according to the 10-20 EEG system. The verum stimulation consisted of 20 min of stimulation with 1 mA direct current, while the placebo stimulation consisted of 32 seconds of stimulation delivered at the beginning of training, with ramp-up/down times of 8 seconds in each case. As described in ref. ^71^, our approach ensured the successful delivery of the stimulation using the StarStim System (Neuroelectrics), and allowed the verification of the completion of training by the immediate transmission of behavioral data into our server.

### Electrophysiology measurements

We conducted a series of electrophysiological investigations to study intracortical inhibition and its modulation during movement preparation using the well-established SICI paradigm^23–25^ in TMS. We performed measurements at rest and during a simple reaction-time task, during which participants had to press a button upon the appearance of a visual cue. We performed these measurements before and after training to look for training- and/or stimulation-related changes in SICI, using the same protocol as in our previous study^4^, targeting the FDI muscle of the left hand. We measured the TMS motor evoked potential using EMG, adjusting the test pulse intensity to 120% resting motor threshold (RMT) and the conditioning pulse intensity to 80% RMT. During the reaction-time task, we applied pulses at 20% and 90% of the time window between the visual cue and the average reaction time.

### Experimental design

**Fig. 6:**
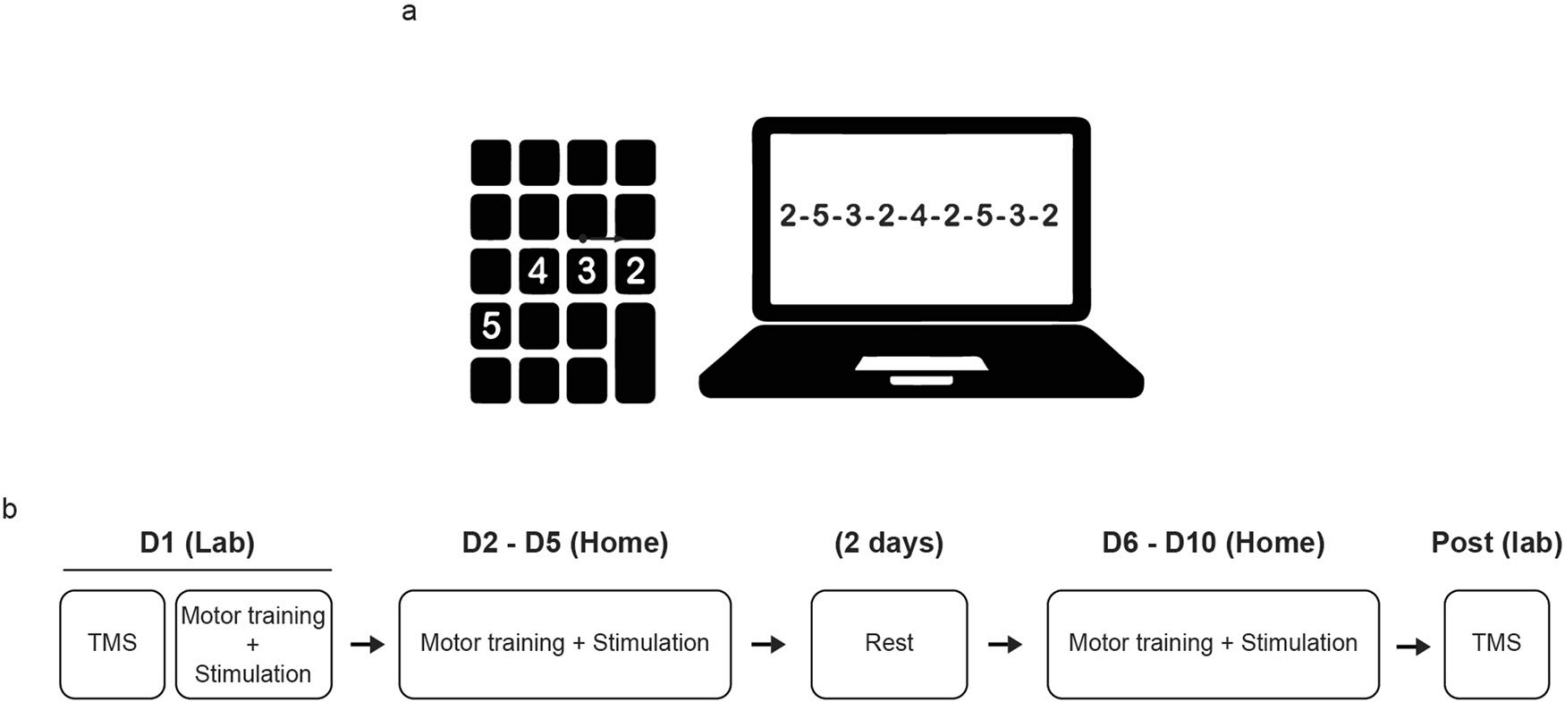
Experimental design. **a** Sequence-tapping task, performed on a laptop using a portable numpad keyboard. **b** Training structure. On the first training day, we started by performing neurophysiological investigations using the SICI paradigm, assumed to reflect GABAergic intracortical inhibition within the motor cortex. Immediately after, we provided a thorough explanation of how to prepare the equipment for motor training and stimulation, as detailed in our previous report (Maceira-Elvira et al., 2020). Participants performed the first training session at our research laboratory and the remaining nine days of training independently at home. After the tenth day of training, participants returned to our laboratory for post-training TMS measurements.

The study followed a randomized, parallel, placebo-controlled, double-blinded design. To facilitate extensive training, taking place over the course of ten days, we asked participants to perform their training with concomitant stimulation at home, for which we provided all the necessary equipment. The participants came for a first visit, during which we explained how to set up the simulation and launch the training at home, providing a detailed manual describing the whole procedure. Participants performed the first training session at our lab, to ensure they had understood the steps involved, and completed the rest of training (i.e., nine days) at home (Fig. 6b).

### Data analysis

We used the same analytical pipeline we implemented for our previous study^4^ to characterize the execution of the sequence for every participant, extracting the temporal execution patterns (i.e., chunking patterns) and quantifying their efficiency. We defined the efficiency of execution in terms of how similar the chunking patterns were to those seen in healthy young adults in our previous study^4^ considering young-like chunking patterns to be more efficient. Please refer to the Supplementary Materials for a detailed account of these analyses.

All statistical tests mentioned throughout the manuscript were performed in R, using the lme4 package^72^ to fit LME models to our data, and the emmeans package^73^ for post hoc testing. We obtained the effect sizes from emmeans, and fitted all models using restricted maximum likelihood. We tested the significance of fixed effects by means of ANOVA Type III on the model using Satterthwaite’s method, and obtained *p* values using the lmerTest package^74^. We performed post hoc tests on significant fixed effects and corrected for multiple comparisons using Tukey’s HSD method^75^. We ran two-tailed post hoc tests on the estimated marginal means (i.e., least-squares means) from our fitted models, with degrees of freedom estimated using the Kenward-Roger method^76^. The present manuscript discusses, with a few exceptions, significant results only (with a cutoff for statistical significance of *p* < 0.05). Please refer to the Report on Statistics (Supplementary Note 10) for the results of all statistical tests. Before running the analyses, single-session data files had to be curated due to technical malfunctions (four participants) and due to non-compliance with the protocol (one participant). Please refer to Supplementary Note 1 for details.

### Optimal/suboptimal learner stratification

The classification of participants as either “optimal” or “suboptimal” learners was based on our previously proposed model^4^, in which the optimal acquisition of the finger-tapping task relies on the prioritization of the improvement of the accuracy at the early stages of training. Based on our observations in various cohorts of healthy young adults, we propose that the optimal acquisition of the finger-tapping task is characterized by the stabilization of the accuracy (i.e., reaching an accuracy plateau) by the end of the first training session. Under this hypothesis, we would consider individuals reaching this plateau on the first training day to be optimal learners, and those reaching the plateau at a later stage to be suboptimal. Following this rationale, we trained a classifier to predict whether participants would reach a plateau in accuracy on the first training day or not based on baseline parameters. Specifically, we used the individual’s age, and their speed and accuracy in the baseline block (performed before training) and the first block of training. Please note that even though we propose age is not the only determining factor in an individual’s ability to acquire new motor skills, we think there are relevant implications to having a certain speed and a certain accuracy, relative to one’s age. In other words, we think there are important differences in being relatively slow and inaccurate at a younger age, compared to being similarly slow and inaccurate at an advanced age. We determined the time point (i.e., the training session) when participants reached a plateau in accuracy by fitting a logarithmic function to the accuracy and detecting the bending point of the curve (please see Supplementary Note 6, and Supplementary fig. 5 for an illustration). We assigned a label of “optimal” to participants whenever a bending point was detected on the first training day, and a label of “suboptimal” otherwise.

We trained the model exclusively on data from a previous study^4^, and we included only participants that had either received no stimulation or placebo stimulation (i.e., we excluded the participants receiving verum stimulation from the training set). We applied a 2nd-degree polynomial expansion to add non-linear relationships among the features, and we used 10-fold cross-validation to select the best model parameters, using 80% of the data for training and 20% for testing in each iteration. After the 10 iterations, we selected the model yielding the highest F1 score, which was a support-vector classifier. After training, we applied the classifier to the training set and extracted the distance of each participant to the hyperplane separating the “optimal” and “suboptimal” classes, and projected these distances on a logistic regression (i.e., sigmoid) function. The sigmoid function has a range between 0 and 1, inclusive, and the interpretation for classification is that values above 0.5 belong to one class, and those below belong to a different class. In the context of our application, participants whose estimated distance from the hyperplane was smaller than 0.5 when being projected onto the sigmoid function were classified as optimal learners, while those above 0.5 were suboptimal. After defining this map, we applied the classifier to the rest of the data (i.e., the data of the present study, with participants receiving either placebo or verum stimulation, and the data of the verum groups in the published dataset) and labeled all participants as either “optimal” and “suboptimal” learners. Further, we segmented each class based on the median of the projected distances, using only the training data. As a result, the optimal and suboptimal classes were split into a higher and a lower tier of learners within each class.

### Reporting summary

Further information on research design is available in the Nature Research Reporting Summary linked to this article.

## Supplementary information

Supplementary materials

Reporting summary

## Data Availability

All data necessary to generate the figures within this manuscript and the Supplementary Materials are available in the Zenodo repository (https://zenodo.org/records/8089332).

## Code availability

The code written to classify individuals into the four groups of learners is available in the Zenodo repository (https://zenodo.org/records/8089332).
