# Supplementary material for: Native learning ability and not age determines the effects of brain stimulation
Source: NPJ Sci Learn. 2024 Nov 27;9:69. doi: 10.1038/s41539-024-00278-y (PMC11603171; doi:10.1038/s41539-024-00278-y)
Supplement: Supplementary file 1 — Supplementary materials [file 41539_2024_278_MOESM1_ESM.pdf]

Title: Native learning ability and not age determines the effects of brain stimulation

Authors: Pablo Maceira-Elvira, Traian Popa, Anne-Christine Schmid, Andéol Cadic-Melchior, Henning Müller, Roger Schaer, Leonardo G. Cohen, Friedhelm C. Hummel

Supplementary materials

Supplementary Note 1. Behavioral data corrections

The participants of the present study performed the motor training and applied stimulation at home, as detailed in our previous report [1]. In a few cases, the participants experienced issues that had to be accounted for before running the analyses detailed in the main text. The problems encountered and the actions we took to account for them are detailed next:

- Participant 217207: The training of this participant was interrupted on two occasions (i.e., on the third block of day 3 and on the third block of day 5) due to stimulation being stopped by the stimulation software. Upon this event, the participant called to get support and was able to resume training. As the score of the blocks in question was affected, we replaced them with the scores of the preceding block (i.e., the second block of the third and the fifth days).
- Participant 217208: On the first block of the first training day, during which the experimenters were present, the participant started training without having connected the keyboard, which resulted in their responses not being recorded by the program. The experimenter noted this problem and added the number of sequences produced while the keyboard was unplugged to the number of sequences detected by the computer. The addition was of three correct sequences.
- Participant 217257: This participant reported doing the last training session (i.e., day 10) while being heavily intoxicated with alcohol. We excluded this training session from the analysis.
- Participant 217309: While performing the training on the third day and during the last two blocks of training, the participant accidentally pressed the “Num Lock” key on the keyboard used to perform the training, which resulted in their responses not being recorded. These two blocks were excluded from the analysis.

- Participant 217357: While performing the training on the second day and during the first two blocks of training, the participant accidentally pressed the “Num Lock” key on the keyboard used to perform the training, which resulted in their responses not being recorded. These two blocks were excluded from the analysis.

Supplementary Note 2. Task scores and the contribution of online/offline learning to total learning

We quantified the general performance of our participants using the same scoring criteria used in our previous study [2]. *Supplementary Figure 1a and 1b* show the scores obtained by our participants, calculated as the number of correct sequences weighted by the ratio of correct to total sequences (i.e., percent correct) generated on each training block. The smaller markers portray individual data, while the larger markers reflect the average for each group, and the error bars represent the standard error of the mean. Please note these scores are not corrected for baseline performance, as we were interested only on the improvement dynamics (i.e., improvement slope) and on the contribution of each acquisition stage to the overall performance change seen towards the end of training.

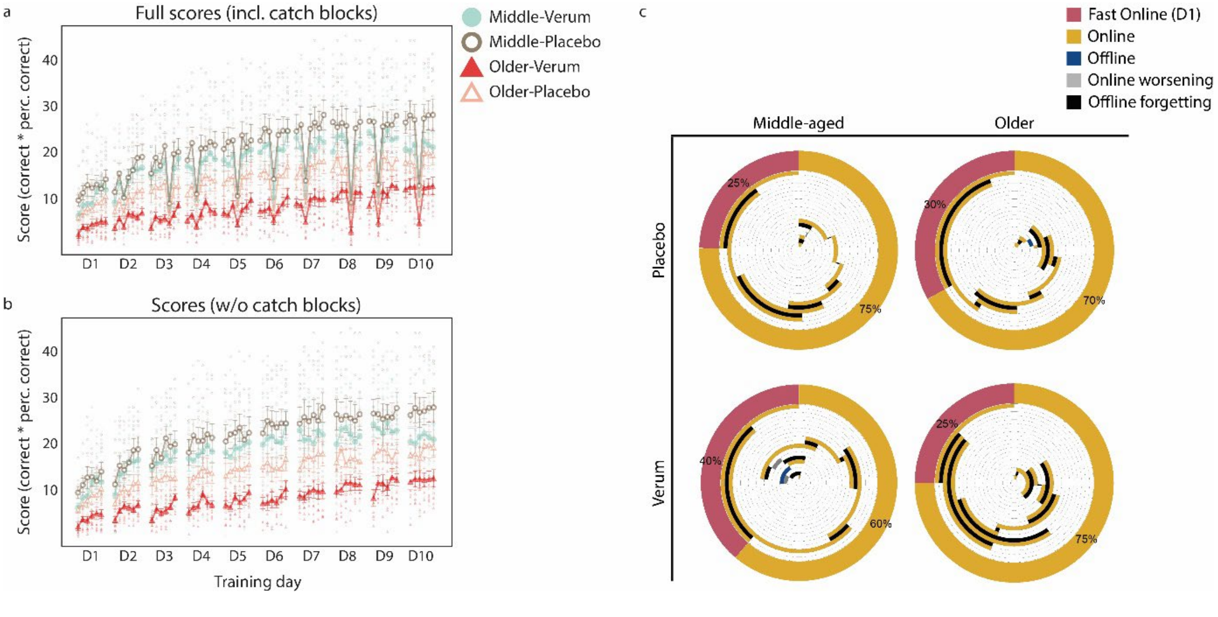

Supplementary Figure 1. Scores obtained by all participants in the finger-tapping task and contribution of the different stages of acquisition to the overall performance change. a. *Participant scores in all blocks within each training session, including the “catch blocks” used to check for generalization of learning. The translucent points depict individual scores, while the opaque points illustrate the mean score of each block in each group. The error bars illustrate the standard error of the mean. Please note the consistent drop in performance within each session, marking the appearance of each catch block.* ***b.*** *Participant scores in all training blocks, excluding the catch blocks.* ***c.*** *Contribution of different stages of learning to total learning over the course of the training. The outermost, thickest ring represents total learning, and is segmented to show the percentage of total learning that was derived from performance changes taking place within the first training session (i.e., fast online learning), performance changes taking place during the training sessions from the second day onwards (i.e., online learning), and performance changes taking place overnight (i.e., offline learning). Please note the absence of offline learning in all groups. The inner circles describe the time course of the different stages of acquisition, in which the last ring corresponds to online learning of the first day (thus matching the outer portion assigned to fast online learning), the one-before-last ring corresponds to performance changes taking place the first and second sessions, and so on.*

Supplementary *Figure 1c* shows the contribution of different stages of acquisition to total learning. Matching our previous findings[2], neither middle-aged nor older adults seem to experience overnight improvements (i.e., offline learning); instead, they seem to worsen overnight (offline forgetting). Please note that the amount of offline forgetting in the group of older adults receiving placebo stimulation resembles that seen in both groups of middle-aged adults, while the group of older adults receiving verum stimulation shows a much more dominant presence of offline forgetting.

Supplementary Note 3. Speed and accuracy in each stimulation group

We studied the effects of applying atDCS over the left-hand representation of the primary motor cortex (M1) during the acquisition of a well-established finger-tapping task [2–6] in middle-aged (50-65 y/o; n = 20, 11 female; ageμ = 59.05 y/o) and older adults (>65 y/o; n = 20, 10 female; ageμ = 71.7 y/o), as they practiced over the course of ten days. The participants received either verum stimulation (i.e., active; middle-aged = 10, ageμ = 58.9; older = 10, ageμ = 71.4) or placebo stimulation (middle-aged = 10, ageμ = 59.2; older = 10, ageμ = 72.1) for 20 minutes daily, as they practiced the finger-tapping task using their left hand. The task consisted in replicating an explicitly shown, nine-digit numerical sequence as fast and as accurately as possible using four buttons. To simplify the report and the discussion of results for each group, we will refer to these groups as "middle-verum", "middle-placebo", "older-verum" and "older-placebo" henceforth. Each training day consisted of seven 90-second practice blocks interleaved with 90 second resting periods; six of the practice blocks contained a fixed sequence (i.e., training sequence), while the seventh contained a different sequence used to assess the generalization of the participants' performance (i.e., "catch blocks"). We applied the same analytical pipeline developed for our previous study [2], describing the performance of our participants in terms of the main drivers for this task (i.e., the speed and the accuracy), and the influence of these parameters in the mechanical execution of the sequence.

Supplementary Figure 2 shows the speed and the accuracy, as well as the dynamics of these two parameters, for the groups of middle-aged and older adults. Please note that these data depict the participants' performance in the trained sequence only (i.e., and not the catch blocks, as there was a consistent drop in performance for these blocks, please see Supplementary *Figure 1a*). Speed (Supplementary *Figure 2a*), quantified as the number of sequences generated by the participants on each training block, was generally higher in the middle-placebo group compared to the older-placebo. However, the statistical testing of this difference showed only a trend towards significance ($F_{[1,18]}$ = 3.12, η^2 = 0.15, P = 0.09), and we did not find evidence of their initial speed being different either (t_{18} = 1.16, d = 0.52, P = 0.25). We did not find evidence for an effect of atDCS on the speed of execution in middle-aged adults, neither in absolute terms ($F_{[1,18]}$ = 0.43, η^2 = 0.02, P = 0.51) nor in the rate of improvement ($F_{[1,1118]}$ = 1.19, η^2 = 0.001, P = 0.27). In older-adults, the older-verum group was initially slower compared to the older-placebo (although the evidence

had only a trend towards significance (t_{12} = 1.84, d = 0.82, P = 0.09)), and the difference in the rate of speed change (i.e., slope) was not statistically significant ($F_{[1,1108]}$ = 0.39, η^2 = 0.0003, P = 0.53). As for the overall speed dynamics (*Supplementary Figure 2c*), the evolution of the speed over the course of training was similar among the groups. As such, these results do not provide evidence for an effect of stimulation on the speed of execution in neither middle-aged nor older adults, matching our previous findings [2].

Supplementary Figure 2b shows the accuracy, calculated as the ratio of correct sequences to total sequences generated by each participant. The middle-placebo group was significantly more accurate than the older-placebo throughout training ($F_{[1,18]}$ = 10.06, η^2 = 0.36, P = 0.005). In middle-aged adults, we did not find the accuracy to be different when comparing both stimulation groups ($F_{[1,18]}$ = 2.97, η^2 = 0.14, P = 0.10). In older adults, the accuracy was significantly higher in the placebo group compared to verum throughout training ($F_{[1,18]}$ = 21.08, η^2 = 0.54, P = 0.0002), starting from the first training block (t_{15} = 3.44, d = 1.54, P = 0.003, Supplementary *Figure 2b*). The slopes were significantly different ($F_{[1,1108]}$ = 9.30, η^2 = 0.008, P = 0.002) between the two stimulation groups, with a steeper improvement of the verum group (t_{1108} = 3.05, d = 13.61, P = 0.002). These differences are illustrated in Supplementary *Figure 2d*. The older-verum group, with lower initial accuracy, improved sharply early in training, reaching a plateau and maintaining similar levels for the remainder of training.

The results obtained from both groups of middle-aged adults and the older-verum group were in correspondence with our previous findings [2]. However, the initial values for speed and accuracy seen in the older-placebo group were higher, more similar to those seen in middle-aged adults. In view of this unexpected difference in baseline performance, we conducted additional comparisons to verify the apparent effect of the stimulation on the accuracy of older adults was not an artifact derived from their initially low skill level, and to better understand the underlying surrogate.

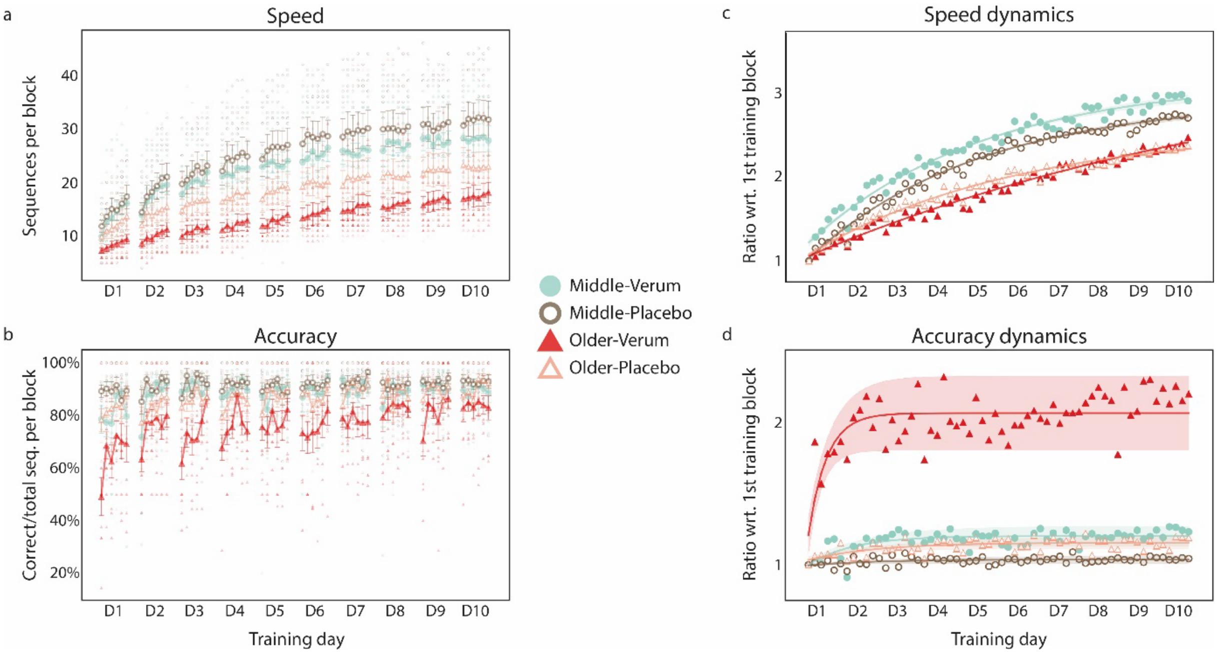

Supplementary Figure 2. Speed and accuracy over training of the sequence-tapping task. a. *Speed in middle-aged and older adults under verum and placebo stimulation, quantified as the total number of generated sequences in each block. The smaller markers portray individual data, while the larger markers reflect the average for each group, and the error bars represent the standard error of the mean.* ***b.*** *Accuracy in the training blocks, calculated as the ratio of correct to total sequences, with the smaller markers portraying individual data and the larger markers reflecting the average for each group. The error bars represent the standard error of the mean.* ***c.*** *Speed dynamics, calculated as the ratio of the average speed of each training block, divided by the average speed of the first block of training within each group. Please note that in spite of the differences in the magnitude of the speed among the age groups, the general improvement follows similar trajectories across all groups.* ***d.*** *Accuracy dynamics, calculated as the ratio of the average accuracy of each training block, divided by the average accuracy of the first block of training within each group. Please note the stark difference between the older-verum, characterized by having initially low accuracies, and the other three groups, starting off at much higher accuracy values. The dynamics in the older-verum group, depicting a sharp increase and stabilization occurring at the early stages of training, match our previous findings in older adults*[2]*. The shaded area represents the 95% confidence interval for the fitted curve.*

Supplementary Note 4. The effect of atDCS in comparably inaccurate individuals

The dynamics we observe in the accuracy of the older-verum group are consistent with our previous findings [2]. However, in that study both the verum and the placebo groups of older adults had similar accuracies initially, whereas in the present study the accuracy in the placebo group was much higher in the beginning. Therefore, a possible explanation for the sharp increase in accuracy in the verum group could be that they started from much lower values and had, thus, more space for improvement. To test whether this was true, we selected individuals from the older-placebo group with initial accuracies within the range of accuracies seen in the older-verum group. The range of accuracies in the first training block of this group went from 14% to 62.5%, excluding one individual with an initial accuracy of 100%, so we considered a range of 0 to 62.5 for this comparison. As there were very few participants with initial accuracies within this range in the older-placebo group in the present study (n = 2), we included individuals from the older-placebo group (n = 4), as well as the unstimulated cohort (n = 5) from our previous study [2]. In total, we compared nine individuals from the older-verum group of this study to eleven older adults under either placebo or no stimulation (older-placebo-unstimulated group). *Supplementary Figure 3* shows the accuracy

dynamics for these two groups, contrasting the sharp increase in accuracy in the older-verum to a gradual increase in the older-placebo-unstimulated group, even though individuals in both groups possessed similar accuracies on the first block of training. For this comparison, we included only the first five days of training of the present study, as in our previous study, participants trained for only five days [2].

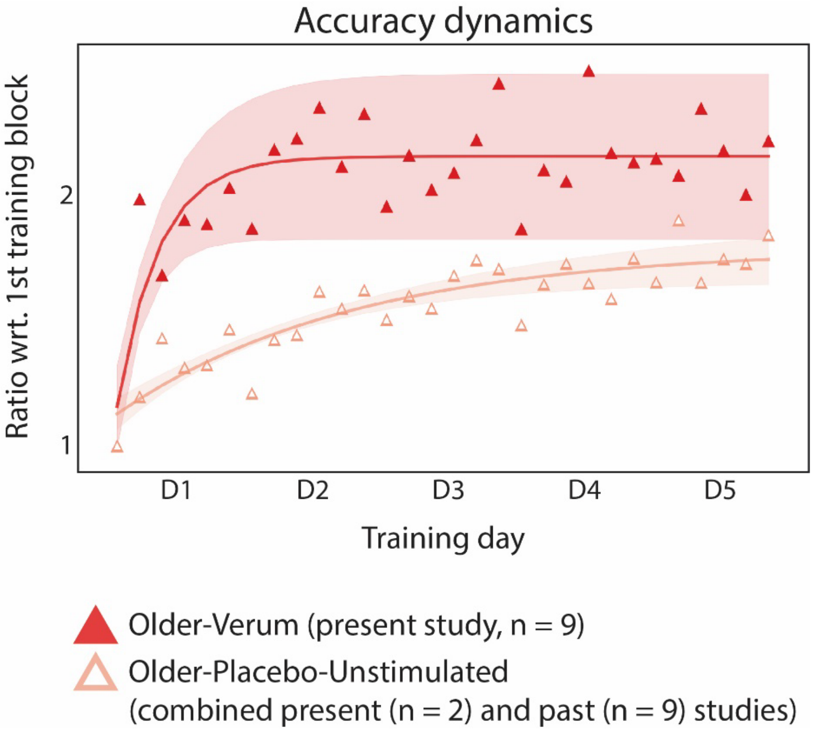

Supplementary Figure 3. Accuracy dynamics in older adults with initial accuracies equal or lower than 62.5%. *We compared individuals from the older-verum group of this study (red, full triangles, n = 9) to individuals from the older-placebo groups of this study (n = 2) and an earlier study (n = 4), as well as unstimulated older individuals from that same study (n = 5)[2] (older-placebo-unstimulated, pink, hollow triangles). Of note is that even though all older adults start off at similar accuracies, the older-verum group experiences a sharp accuracy increase on the first training day and reaches a plateau, while the older-placebo-unstimulated group increases their accuracy gradually over the course of training. The shaded area represents the 95% confidence interval for the fitted curve. Please note there are only five days of training displayed, as opposed to the ten days of training shown in Supplementary Figure 2. The reason is that in the past studies we conducted, participants were required to practice for five days instead of ten.*

Supplementary Note 5. Streamlining the mechanical execution of the motor sequence

In a previous study [2], we found that optimizing the accuracy at the early stages of training enabled the streamlining of the execution of the sequence, leading to the generation of efficient temporal patterns commonly referred to as motor chunks [7–9]. In turn, the generation of efficient chunking patterns seemed to depend on the prime placed on speed, with a higher prime leading to an earlier formation of chunking patterns, and a lower prime resulting in such patterns appearing later in training. Based on those observations, we expected individuals from the older-placebo group of the present study to generate efficient chunking patterns sooner than those in the older-verum group, as they were significantly more accurate and faster from the beginning. *Supplementary Figure 4* shows the efficiency of generated chunking patterns by all

individuals of this study. We quantified the efficiency in the same way and using the same model as in our previous study [2].

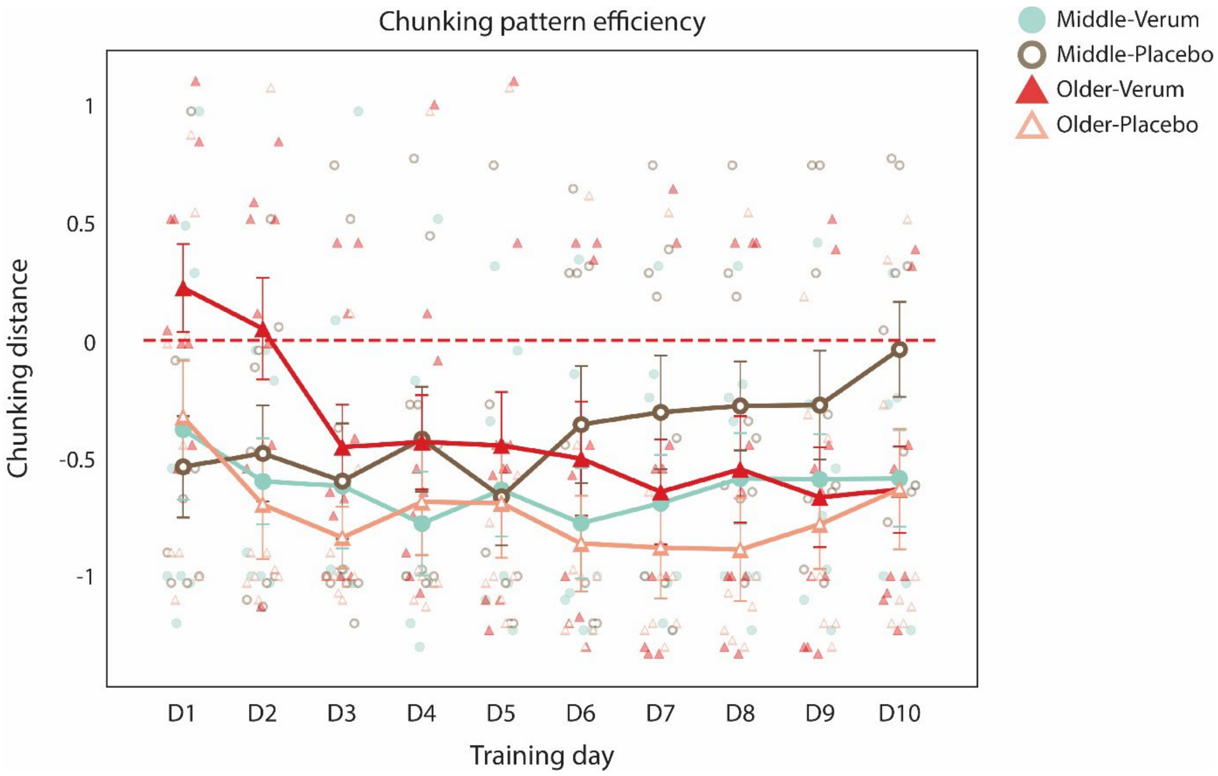

Supplementary Figure 4. Chunking pattern efficiency. *Evolution of the chunking patterns generated by all participants over the course of training. The measure of efficiency is the "chunking distance" described in a previous paper[2], which values represent more efficient (i.e., negative values) or less efficient (i.e., positive values) chunking patterns emerging during the execution of the finger-tapping task. The smaller markers depict the values assigned to each individual on each day of training, while the larger markets illustrate the average values per day. The error bars represent the standard error of the mean. Please note that most individuals in both groups of middle-aged adults and the older-placebo group, possessing relatively high speeds and accuracies at the beginning of training (see Supplementary Figure 2a and 2b), generate efficient chunking patterns on the first training day, which do not seem to vary much over the remainder of training. In contrast the older-verum group, with initially lower speed and accuracy compared to the other three groups, generate efficient chunking patterns at a later stage during training. However, matching our previous results[2], half of the participants in this group generate efficient chunking patterns on the first day, with most of the others doing so by the third day.*

Our results show that almost all individuals from the middle-verum, middle-placebo and older-placebo groups generated efficient chunking patterns from the first day of training. This meets our expectations, as the three groups had initially high accuracy and speed. In the older-verum group, about half of the participants generated efficient patterns on the first training day, with most of them doing so by the third day of training; this is the same behavior we found in our previous study for the older-verum [2].

Supplementary Note 6. "Optimal" and "suboptimal" label creation

The definition we used for optimal and suboptimal learners was based on our previous findings [2], in which the groups of individuals acquiring the finger-tapping task most efficiently where able to optimize their accuracy at the early stages of training (i.e., by the end of the first day). Accordingly, we estimated the time point at which individuals reached a plateau in their accuracy. Please note this was not necessarily at 100%;

indeed, a stable accuracy at 100% was rare. Rather, the participants seem to settle for an "acceptable" error rate, around which their performance appears to stabilize. *Supplementary Figure 5* shows an example of two participants, with IDs 115104 (*Supplementary Figure 5a*) and 115304 (*Supplementary Figure* 5b); these participants to a previously published dataset [2], in which participants practiced over the course of five consecutive days. In the figure, we show the accuracy values per block of training, as well as a logarithmic function fitted to these values. The red, dashed lines indicate the estimated stabilization value for the accuracy (horizontal), and the block at which the fitted line describes the bend or the "knee" [10] of the fitted line (vertical). The point where these two lines intersect marks the knee point for the fitted function, and constitutes the block at which the accuracy stabilizes. In the context of our interpretation, when this bend occurs within the first 6 training blocks (i.e., the first training day), participants are able to acquire the finger-tapping task most efficiently.

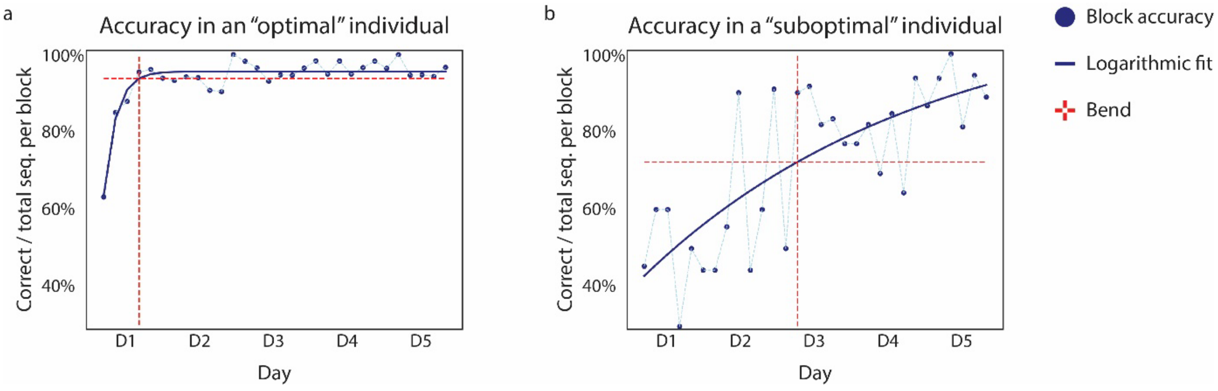

Supplementary Figure 5. Label assignment for optimal and suboptimal individuals based on the estimated accuracy stabilization time point. *Estimation of the block at which the accuracy stabilizes based on the knee point of a logarithmic function adjusted to the accuracy of each block. The blue dots represent the accuracy on each training block, while the blue, solid line illustrates the fitted function. The red, dashed lines represent the value around which the accuracy was estimated to settle (horizontal) and the training block when this occurred (vertical).* ***a.*** *Accuracy in an "optimal" learner, whose accuracy stabilized by the end of the first training session.* ***b.*** *Accuracy in a "suboptimal" learner, whose accuracy stabilized halfway through training.*

Supplementary Note 7. Neurophysiological investigations on GABAergic intracortical inhibition at rest and during movement preparation

Supplementary Figure 6 shows the SICI measurements at rest (i.e., SICIrest, (a)) and during movement preparation (i.e., SICImodulation, (b)), averaged per stimulation group. The SICI ratio variable was calculated by dividing the average MEP amplitude obtained when applying the SICI paradigm by the average MEP amplitude obtained when applying the test pulse. The test pulse was delivered at an intensity close to 120% of the resting motor threshold (i.e., RMT), but was adjusted to obtain an MEP amplitude of 1 mV in 5 out of 10 consecutive single pulses. Modulation was calculated

as the difference in MEP amplitude when the SICI paradigm was delivered at 90% and 20% of reaction time, as measured during a simple reaction time task.

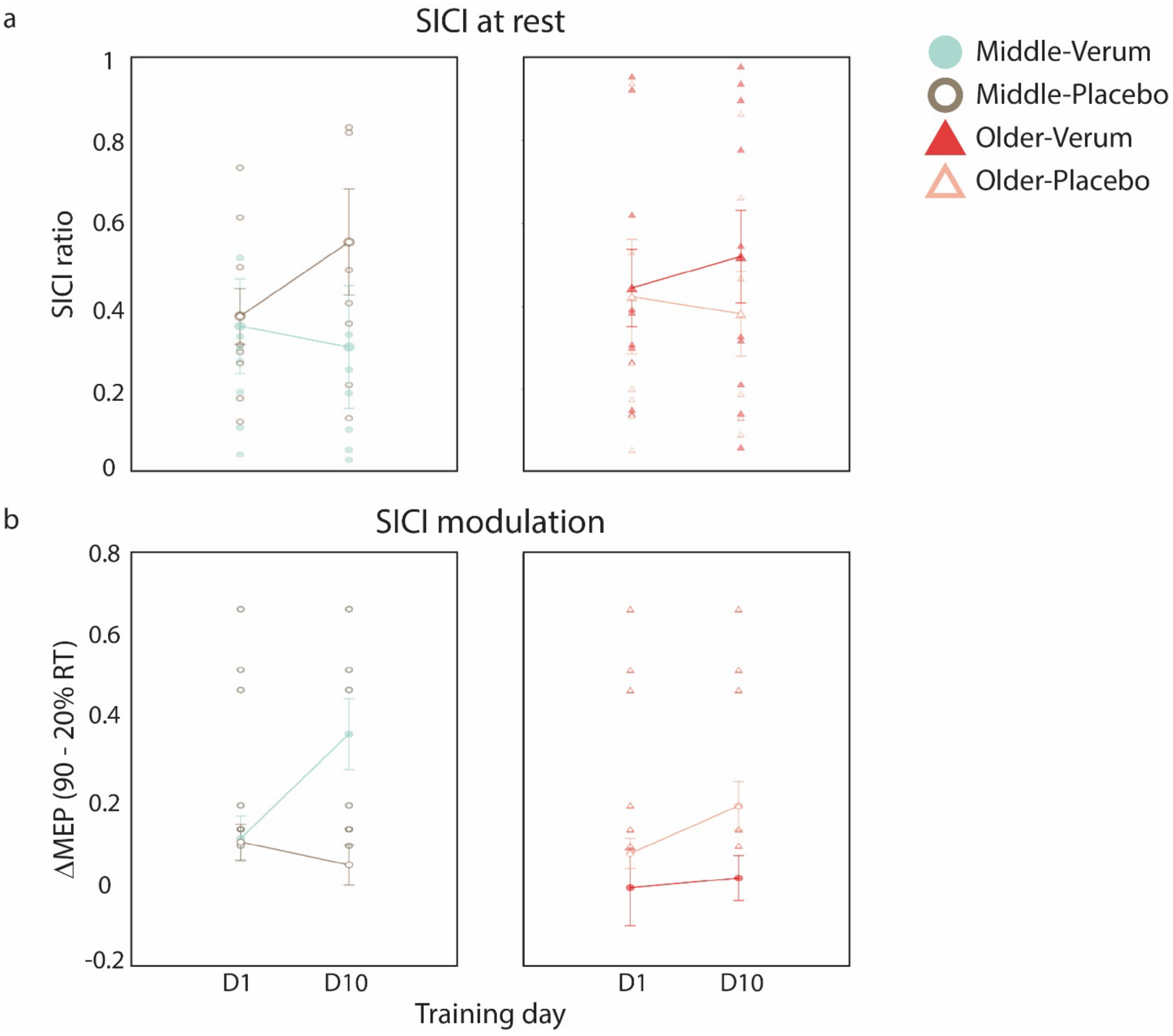

*Supplementary Figure 6. **GABAergic intracortical inhibition measured at M1 using the SICI TMS paradigm. a.** SICI recordings measured while participants were at rest and **b.** performing a reaction-time task. GABAergic intracortical inhibition was estimated as the ratio between the motor evoked potential (MEP) amplitude in SICI trials to the amplitude obtained when applying the test stimulus alone (calibrated to ~1 mV). Modulation was calculated as the difference in SICI ratio when applying the SICI paradigm at 90% of reaction time with respect to applying the SICI paradigm at 20% of reaction time. The translucent markers depict individual data points, while the solid markers represent the average per age and per stimulation group. The error bars represent the standard error of the mean.*

Supplementary Note 8. Speed dynamics in the Modeling and Validation datasets

We found atDCS to have an effect on the accuracy and not on the speed of execution. As further verification, we compared the change in speed (i.e., the speed dynamics) in the four learner categories, for which we reported marked differences in terms of accuracy in the main text. As shown in *Supplementary Figure 7*, the speed dynamics were similar among groups. Most statistical comparisons did not provide evidence for significant differences related to stimulation in the speed dynamics, with a few

exceptions: in the low tier of the suboptimal learners in the Modeling dataset (*Supplementary Figure 7a*), the change in speed was significantly larger in the verum group with respect to the placebo on day 2 (t_{52} = 3.079, d = 1.864, P = 0.003) and on day 5 (t_{52} = 2.843, d = 1.721, P = 0.0064). In the low tier of optimal learners of the Validation dataset (*Supplementary Figure 7b*), the change in speed in the verum group was significantly higher on day 1 (t_{16} = 2.118, d = 1.675, P = 0.049) and day 2 (t_{16} = 2.876, d = 2.274, P = 0.01).

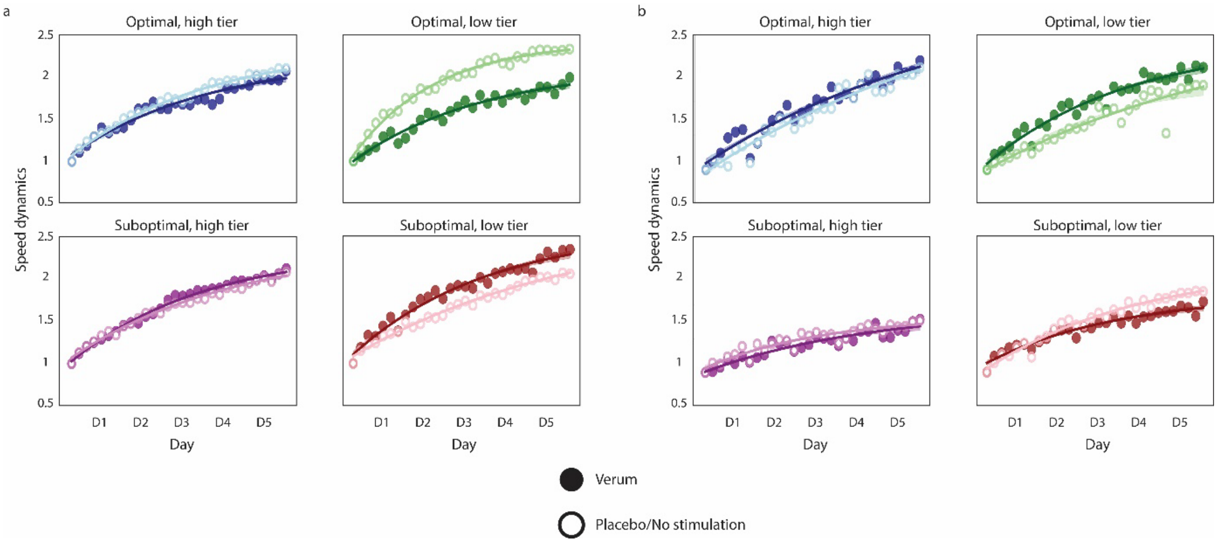

Supplementary Figure 7. Speed dynamics for the different learner tiers. *Speed dynamics, calculated as the ratio of the average speed of each training block, divided by the average speed of the first block of training within each group.* ***a.*** *Speed dynamics in the Modeling dataset.* ***b.*** *Speed dynamics in the Validation dataset. Please refer to the main text for details on the number and age of the participants assigned to each group.*

Supplementary Note 9. Relationship between the MMSE scores and the estimated likelihood to respond to stimulation

One of the inclusion criteria we applied for volunteers to be enrolled as healthy participants into our study consisted in an assessment of their cognitive functions using the Mini-Mental State Examination (MMSE, [11]). This test was only applied to middle-aged and older participants to assert the preservation of cognitive functions in these individuals. Thus, the MMSE was intended to ensure all participants possessed well-preserved cognitive functions, requiring at least 26 out of 30 points for enrolment.

The method we propose involves the characterization of an individual's ability to integrate task relevant information efficiently at the early stages of training. As such, we decided to test the relationship between the estimated likelihood to benefit from stimulation, and the MMSE scores in middle aged and older adults. *Supplementary Figure 8* shows the results, which do not suggest any relationship between the MMSE scores and the estimated likelihoods.

All individual scores shown in *Supplementary Figure 8* are available in the Zenodo repository https://zenodo.org/records/8089332. A minimum score of 26 was asserted for all participants, as it was an inclusion criterion for our study. However, the paper

assessment forms of nine participants were not accessible any more (five middle-aged, four older); these scores are missing and could not be included in this analysis..

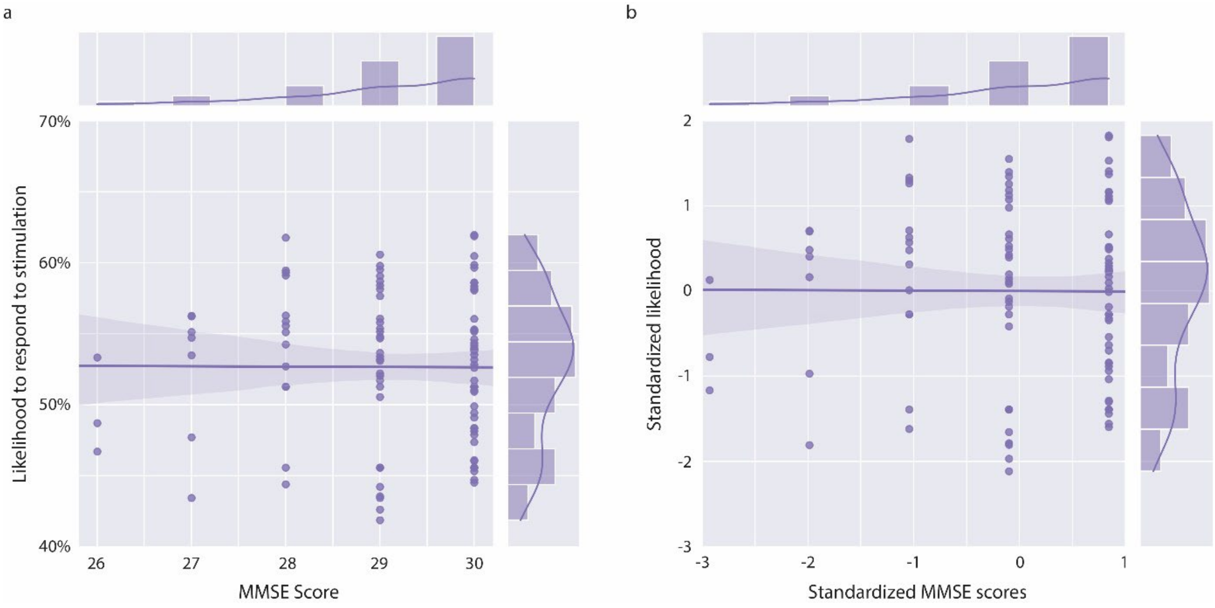

Supplementary Figure 8. Relationship between MMSE scores and estimated likelihood to benefit from stimulation in middle-aged and older adults. a. *MMSE scores plotted against the estimated likelihood to benefit from stimulation. The inclusion criterion related to the MMSE required participants to have at least 26 out of 30 points, with average scores of 29.1 (std = 0.76, median = 29).* ***b.*** *Standardized scores and likelihoods, centered to remove the order of magnitude-related bias from our comparison. Please note that our results do not suggest a relationship exists between the score obtained in the MMSE and the estimated likelihood to benefit from stimulation.*

Supplementary Note 10. Report on statistical testing

As a complement to this document, we have generated a R notebook in HTML format detailing all the statistical tests we conducted, leading to the results detailed in the main text. This notebook is stored in the Zenodo repository https://zenodo.org/records/8089332.

Supplementary Note 11. Adverse events related to the application of stimulation

Three participants reported mild adverse effects related to the stimulation techniques we used.

- Participant 216258: Mild headache after neurophysiological measurement (TMS)
- Participant 217257: Mild headache lasting a few minutes after tDCS + motor training sessions
- Participant 217360: Skin redness after applying tDCS

Supplementary references

1. Maceira-Elvira, P., Popa, T., Schmid, A.-C. & Hummel, F. C. Feasibility of home-based, self-applied transcranial direct current stimulation to enhance motor learning in middle-aged and older adults. *Brain Stimulation* **13**, 247–249 (2020).
2. Maceira-Elvira, P. *et al.* Dissecting motor skill acquisition: Spatial coordinates take precedence. *Sci. Adv.* **8**, eabo3505 (2022).
3. Draaisma, L. R., Wessel, M. J., Moyne, M., Morishita, T. & Hummel, F. C. Targeting the frontoparietal network using bifocal transcranial alternating current stimulation during a motor sequence learning task in healthy older adults. *Brain Stimulation* **15**, 968–979 (2022).
4. Walker, M. P., Brakefield, T., Allan Hobson, J. & Stickgold, R. Dissociable stages of human memory consolidation and reconsolidation. *Nature* **425**, 616–620 (2003).
5. Wessel, M. J. *et al.* Multifocal stimulation of the cerebro-cerebellar loop during the acquisition of a novel motor skill. *Sci Rep* **11**, 1756 (2021).
6. Zimerman, M. *et al.* Neuroenhancement of the aging brain: Restoring skill acquisition in old subjects. *Annals of Neurology* **73**, 10–15 (2013).
7. Rosenbaum, D. A., Cohen, R. G., Jax, S. A., Weiss, D. J. & Van Der Wel, R. The problem of serial order in behavior: Lashley's legacy. *Human Movement Science* **26**, 525–554 (2007).
8. Sakai, K., Kitaguchi, K. & Hikosaka, O. Chunking during human visuomotor sequence learning. *Experimental Brain Research* **152**, 229–242 (2003).
9. Verwey, W. B. Buffer Loading and Chunking in Sequential Keypressing. *J. Exp. Psychol. Hum. Percept. Perform.* **22**, 544 (1996).
10. Satopaa, V., Albrecht, J., Irwin, D. & Raghavan, B. Finding a 'Kneedle' in a Haystack: Detecting Knee Points in System Behavior. in *2011 31st International Conference on Distributed Computing Systems Workshops* 166–171 (IEEE, Minneapolis, MN, USA, 2011). doi:10.1109/ICDCSW.2011.20.
11. Folstein, M. F., Robins, L. N. & Helzer, J. E. The mini-mental state examination. *Archives of general psychiatry* **40**, 812–812 (1983).
