# Supplementary material for: Native learning ability and not age determines the effects of brain stimulation
Source: NPJ Sci Learn. 2024 Nov 27;9:69. doi: 10.1038/s41539-024-00278-y (PMC11603171; doi:10.1038/s41539-024-00278-y)
Supplement: Supplementary file 2 — Reporting summary [file 41539_2024_278_MOESM2_ESM.pdf]

nature portfolio

Corresponding author(s): Friedhelm C. Hummel

Last updated by author(s): Jun 25, 2024

Reporting Summary

Nature Portfolio wishes to improve the reproducibility of the work that we publish. This form provides structure for consistency and transparency in reporting. For further information on Nature Portfolio policies, see our Editorial Policies and the Editorial Policy Checklist.

Statistics

For all statistical analyses, confirm that the following items are present in the figure legend, table legend, main text, or Methods section.

n/a	Confirmed	
☐	☒	The exact sample size (*n*) for each experimental group/condition, given as a discrete number and unit of measurement
☐	☒	A statement on whether measurements were taken from distinct samples or whether the same sample was measured repeatedly
☐	☒	The statistical test(s) used AND whether they are one- or two-sided<br>*Only common tests should be described solely by name; describe more complex techniques in the Methods section.*
☐	☒	A description of all covariates tested
☐	☒	A description of any assumptions or corrections, such as tests of normality and adjustment for multiple comparisons
☐	☒	A full description of the statistical parameters including central tendency (e.g. means) or other basic estimates (e.g. regression coefficient) AND variation (e.g. standard deviation) or associated estimates of uncertainty (e.g. confidence intervals)
☐	☒	For null hypothesis testing, the test statistic (e.g. *F*, *t*, *r*) with confidence intervals, effect sizes, degrees of freedom and *P* value noted<br>*Give P values as exact values whenever suitable.*
☒	☐	For Bayesian analysis, information on the choice of priors and Markov chain Monte Carlo settings
☐	☒	For hierarchical and complex designs, identification of the appropriate level for tests and full reporting of outcomes
☐	☒	Estimates of effect sizes (e.g. Cohen's *d*, Pearson's *r*), indicating how they were calculated

Our web collection on statistics for biologists contains articles on many of the points above.

Software and code

Policy information about availability of computer code

Data collection	The finger-tapping task: In-house task written in javascript and deployed on a tablet. TMS recordings: Signal by CED (https://ced.co.uk/products/sigovin)
Data analysis	Statistical testing was done using R (https://www.r-project.org/) and machine learning using Python's SKlearn toolbox (https://scikit-learn.org/stable/). The code is available on the Zenodo repository (https://zenodo.org/records/8089333)

For manuscripts utilizing custom algorithms or software that are central to the research but not yet described in published literature, software must be made available to editors and reviewers. We strongly encourage code deposition in a community repository (e.g. GitHub). See the Nature Portfolio guidelines for submitting code & software for further information.

Data

Policy information about availability of data

All manuscripts must include a data availability statement. This statement should provide the following information, where applicable:

- Accession codes, unique identifiers, or web links for publicly available datasets
- A description of any restrictions on data availability
- For clinical datasets or third party data, please ensure that the statement adheres to our policy

The datasets are available on the Zenodo repository (https://zenodo.org/records/8089333)

Research involving human participants, their data, or biological material

Policy information about studies with human participants or human data. See also policy information about sex, gender (identity/presentation), and sexual orientation and race, ethnicity and racism.

Reporting on sex and gender	We only specify the number of male and female participants in each cohort, and only to show the groups are balanced.
Reporting on race, ethnicity, or other socially relevant groupings	We do not use any such variables.
Population characteristics	40 healthy adults volunteered specifically for this study. Middle-aged (50-65 y/o; n = 20, 11 female; mean age = 59.1 y/o), and older (>65 y/o; n = 20, 10 female; mean age = 71.7 y/o) adults
Recruitment	We distributed information forms containing a general description of the aim of the study and the techniques involved, and waited to be contacted by interested individuals.
Ethics oversight	Ethics committee Vaud, Switzerland (project No. 2017-00301)

Note that full information on the approval of the study protocol must also be provided in the manuscript.

Field-specific reporting

Please select the one below that is the best fit for your research. If you are not sure, read the appropriate sections before making your selection.

☒ Life sciences ☐ Behavioural & social sciences ☐ Ecological, evolutionary & environmental sciences

For a reference copy of the document with all sections, see nature.com/documents/nr-reporting-summary-flat.pdf

Life sciences study design

All studies must disclose on these points even when the disclosure is negative.

Sample size	We based the sample size calculation on the effect sizes of two previous studies from our group, specifically Zimerman et al., 2013 (standardized mean difference 1.33) and Heise et al., 2014 (standardized mean difference 0.53) and aimed for the average of both (0.93).
Data exclusions	Data exclusions are described in Supplementary materials (1 training session for one subject because the subject performed home-based training while intoxicated with alcohol, and 2 training blocks for two subjects due to technical problems with the keyboard).
Replication	We replicated the effects seen in different learner tiers among participants of a previously published study (Maceira-Elvira et al., 2022. Science Advances), in a new cohort of 40 healthy volunteers.
Randomization	A member of our group who was not involved in the study in any way created a list with codes for each age group, with either verum or placebo stimulation assigned to them. The study participants were assigned codes sequentially as they enrolled in the study.
Blinding	All investigators involved in either the collection or the processing of the data, or on any other aspect of the study, were blinded to the stimulation assigned to each participant code during data acquisition and the implementation of processing pipelines.

Reporting for specific materials, systems and methods

We require information from authors about some types of materials, experimental systems and methods used in many studies. Here, indicate whether each material, system or method listed is relevant to your study. If you are not sure if a list item applies to your research, read the appropriate section before selecting a response.

Materials & experimental systems

n/a	Involved in the study	
☒	☐	Antibodies
☒	☐	Eukaryotic cell lines
☒	☐	Palaeontology and archaeology
☒	☐	Animals and other organisms
☒	☐	Clinical data
☒	☐	Dual use research of concern
☒	☐	Plants

Methods

n/a	Involved in the study	
☒	☐	ChIP-seq
☒	☐	Flow cytometry
☒	☐	MRI-based neuroimaging

Plants

Seed stocks	*Report on the source of all seed stocks or other plant material used. If applicable, state the seed stock centre and catalogue number. If plant specimens were collected from the field, describe the collection location, date and sampling procedures.*
Novel plant genotypes	*Describe the methods by which all novel plant genotypes were produced. This includes those generated by transgenic approaches, gene editing, chemical/radiation-based mutagenesis and hybridization. For transgenic lines, describe the transformation method, the number of independent lines analyzed and the generation upon which experiments were performed. For gene-edited lines, describe the editor used, the endogenous sequence targeted for editing, the targeting guide RNA sequence (if applicable) and how the editor was applied.*
Authentication	*Describe any authentication procedures for each seed stock used or novel genotype generated. Describe any experiments used to assess the effect of a mutation and, where applicable, how potential secondary effects (e.g. second site T-DNA insertions, mosiacism, off-target gene editing) were examined.*
